# Inhibitory Effect of Alnustone on Survival and Lung Metastasis of Colorectal Cancer Cells

**DOI:** 10.3390/nu16213737

**Published:** 2024-10-31

**Authors:** Shin-Young Park, Jeong-Geon Mun, Yoon-Seung Lee, Sun-Bin Lee, Su-Jin Kim, Jeong-Ho Jang, Ho-Yoon Kim, Seung-Heon Hong, Ji-Ye Kee

**Affiliations:** Department of Oriental Pharmacy, College of Pharmacy, Wonkwang-Oriental Medicines Research Institute, Wonkwang University, 460 Iksandae-ro, Iksan 54538, Jeonbuk, Republic of Korea; anddy11@wku.ac.kr (S.-Y.P.); wjdrjs92@daum.net (J.-G.M.); yslee308@wku.ac.kr (Y.-S.L.); binhyung2@naver.com (S.-B.L.); u_u0409@naver.com (S.-J.K.); vuwo0522@gmail.com (J.-H.J.); windbell2574@naver.com (H.-Y.K.)

**Keywords:** alnustone, apoptosis, autophagy, cell cycle arrest, colorectal cancer, lung metastasis

## Abstract

Background/Objectives: Alnustone (Aln) is an effective compound of *Alpinia katsumadae* Hayata. Aln possesses various pharmacological activities such as antibacterial, anti-inflammatory, and anti-cancer effects. However, the inhibitory effect of Aln on colorectal cancer (CRC) has not yet been identified. Thus, research was conducted to clarify whether Aln can suppress the proliferative and metastatic ability of CRC cells. Methods: A cell viability assay was performed to confirm the decrease in CRC cell viability following Aln treatment. Flow cytometry was carried out to evaluate the effects of Aln on cell cycle arrest, autophagy, and apoptosis in CRC cells. In addition, a lung metastasis animal model was used to check the inhibitory effect of Aln on the metastasis of CRC cells. Results: Aln remarkably diminished the viability and colony-forming ability of several CRC cell lines. In addition, Aln led to a halt at the G0/G1 phase through downregulating cyclin D1-CDK4 in CRC cells. The upregulation of LC3B and p62 expression by Aln triggered autophagy of CRC cells. Moreover, Aln promoted mitochondrial depolarization, resulting in apoptosis of CRC cells. Oral administration of Aln significantly restrained the metastasized lung tumor nodules. Conclusions: This study demonstrated that Aln can suppress the survival and lung metastasis of CRC cells by promoting cell cycle arrest, autophagy, and apoptosis.

## 1. Introduction

Among cancers, colorectal cancer (CRC), which occurs in the colon or rectum, is the third most prevalent cancer globally in 2020, and the second highest leading cause of cancer mortality [1]. The 5-year survival rate stands at 15% when cancer has spread to distant sites or lymph nodes. In addition, about 22% of patients receive a diagnosis at this late stage [2]. Metastases are diagnosed in about 20% of CRC patients [3]. Even in patients initially confined to the large intestine, up to 50% develop metastasis. Metastasis of colon cancer mainly occurs in the liver and lungs [4].

The cell cycle is a sequence of events necessary for proper cell division, playing a decisive role in cell growth and proliferative activity. The disruption of cell cycle regulation results in abnormal cell proliferation, contributing to cancer development [5]. In cell cycle regulation, cyclin-dependent protein kinase (CDK) forms a functional unit by combining with cyclin. Cyclin and CDK are selectively expressed during distinct stages of the cell cycle, thereby creating particular cyclin–CDK complexes that become activated at each cell cycle stage [6]. Thus, controlling the cyclin–CDK complex to induce cell cycle arrest is a common approach in cancer therapy [7].

Autophagy refers to a mechanism that degrades unnecessary or malfunctioning organelles in cells and reproduces them as energy sources to maintain homeostasis and promote differentiation, development, and survival [8]. It has been studied that autophagy functions as a suppressor by degrading potential oncogenic molecules in the early stages of tumorigenesis [9]. Additionally, there is evidence that excessive or defective autophagy processes may trigger autophagic cell death, according to the specific circumstances of cancer progression [10]. Microtubule-associated protein 1 light chain 3 (LC3) plays a crucial role in the formation of autophagosomes, with LC3-I conjugated to phosphatidylethanolamine and thereby converted into LC3-II, a key component of the autophagosome membrane. LC3B is one of the mammalian isoforms of LC3 [11]. p62 (SQSTM1) is a crucial adaptor protein in autophagy, functioning as a cargo receptor that binds to ubiquitinated proteins and directs them to autophagosomes for degradation [12].

Apoptosis, called programmed cell death, is a controlled mechanism that plays a fundamental role in maintaining multicellular life [13]. Apoptosis can broadly proceed through extrinsic and intrinsic pathways. Intracellular stress, such as hypoxia, oncogenes, and direct DNA damage, has the potential to trigger the intrinsic apoptotic pathway, which is described as the mitochondrial pathway [14,15]. In the process of apoptosis, there is a reduction in the mitochondrial transmembrane potential, which is involved with mitochondrial permeability and pore opening. Thus, cytochrome C is released from mitochondria, inducing apoptosome formation. This complex activates procaspase-9, which is an initiator, and then cleavage of the executioner poly ADP-ribose polymerase (PARP) and caspase-3 is activated, resulting in cell apoptosis [16].

*Alpinia katsumadae* Hayata (*Alpinia katsumadai* Hayata), belonging to the family Zingiberaceae, is an aromatic herb utilized for the therapy of digestive problems by removing dampness-caused phlegm in the digestive organ and resolving qi stagnation with pungent, warm therapies in oriental medicine [17]. Alnustone (Aln) is a nonphenolic diarylheptanoid (C_19_H_18_O) of the ripened seeds of *A. katsumadae* [17,18]. This compound has been shown to exhibit varied pharmacological activities, including anti-inflammatory, antiviral, and antibacterial effects [19,20,21]. Also, Aln shows a suppressive effect on hepatocellular carcinoma cells in in vitro and in vivo experiments [22]. Since there are no reports on the suppressive effect of Aln on CRC, several experiments were conducted to investigate and clarify its impacts on CRC cell inhibition.

## 2. Materials and Methods

### 2.1. Reagents and Antibodies

Aln (purity > 98%) and 5-fluorouracil (5-FU) were purchased from Chengdu Biopurify Phytochemicals Ltd. (Chengdu, China) and Sigma-Aldrich (Burlington, MA, USA), respectively. Dulbecco’s modified Eagle’s medium (DMEM), RPMI 1640, fetal bovine serum (FBS), and penicillin–streptomycin (10,000 U/mL) were purchased from Thermo Fisher Scientific (Waltham, MA, USA). Antibodies against LC3B, p62, caspase-3, caspase-9, PARP, cytochrome C, phospho-p38, phospho-ERK, phospho-AMPK, and AMPK were purchased from Cell Signaling Technology (Danvers, MA, USA). Anti-cyclin B1, CDK1, cyclin D1, CDK4, p38, ERK, GAPDH, α-tubulin, and β-actin antibodies were purchased from Santa Cruz Biotechnology (Dallas, TX, USA).

### 2.2. Cell Culture

The murine (CT26 and MC38) and human (HCT116 and SW620) CRC cell lines were obtained from the Korean Cell Line Bank (Seoul, Republic of Korea). CT26 and MC38 cells were cultured in DMEM, while HCT116 and SW620 cells were grown in RPMI. All media were supplemented with penicillin (100 units/mL), streptomycin (100 µg/mL), and 10% heat-inactivated FBS. All cell lines were maintained at 37 °C in the atmosphere of a 5% CO_2_ incubator.

### 2.3. Animals

Five-week-old BALB/c mice (female, 17–18 g) were acquired from Samtako (Osan, Republic of Korea). The mice were housed in cages in the following five groups (*n* = 6 per group): the control group, three Aln treatment groups (3.125, 6.25, and 12.5 mg/kg), and the 5-FU treatment group (10 mg/kg). Throughout the experiment, the mice were accommodated in a laminar flow system operating room, where we maintained a half-day light/dark cycle, and the temperature (22 ± 1 °C) and humidity (55 ± 1%) remained constant. The in vivo experiment was carried out under accepted international principles for the care and use of laboratory animals, following Wonkwang University guidelines (WKU23-22) and in compliance with the ARRIVE guidelines.

### 2.4. Assays of Cell Viability

The D-Plus™ CCK cell viability assay kit (Dongin LS, Seoul, Republic of Korea) was employed to estimate the number of viable cells. CT26 and MC38 cells (5 × 10^3^ cells/well) and HCT116 and SW620 cells (1 × 10^4^ cells/well) were spread in 96-well plates and cultured overnight before exposure to Aln (20–80 µM). After incubation for 24 h, the supernatants of each well were replaced with a mixture of CCK solution and fresh medium (1:9). Afterwards, a microplate reader (Molecular Devices, San Jose, CA, USA) was used to measure the absorbance at 450 nm.

### 2.5. Colony Formation Assay

CT26 (5 × 10^2^ cells/well) and HCT116 (2 × 10^3^ cells/well) cells were seeded in a 12-well culture plate. Following an overnight period of cell stabilization, the cells were subjected to incubation with Aln (20–80 μM) for 7 days. Then, 10% formaldehyde was used to fix the colonies for 15 min, and the colonies were washed with phosphate-buffered saline (PBS). Crystal violet solution was appended to stain the colonies for 30 min, which were washed with PBS 3 times. The colonies were dried overnight. Subsequently, the stained colonies were dissolved in methanol, and a microplate reader measured the absorbance to calculate the relative level of colony formation.

### 2.6. Cell Cycle Analysis

The distribution of cell cycle phases was measured using the Muse^®^ Cell Cycle Kit (Luminex, Austin, TX, USA) depending on the manufacturer’s protocol. Briefly, CT26 cells (5 × 10^3^ cells/well) and HCT116 cells (1 × 10^4^ cells/well) were spread in a 96-well culture plate and stabilized overnight. The cells were treated with Aln (20–80 µM) for 24 h. The harvested cells were suspended again in 100 μL of Muse^TM^ Cell Cycle Reagent and incubated for 20 min at room temperature with the light blocked. The Muse^TM^ Cell Analyzer was employed to measure cell cycle arrest. Quantification of the percentage of cell cycle phase distribution was performed using Muse analysis software (version 1.9, Millipore, MA, USA).

### 2.7. Autophagy Detection

Autophagy was assessed using the Muse^®^ Autophagy LC3-antibody-based Kit (Luminex, Austin, TX, USA) according to the manufacturer’s instructions. CT26 and HCT116 cells (1 × 10^4^ cells/well) were spread in a 96-well culture plate, stabilized overnight, and then exposed to Aln (20–80 µM) for 24 h. Aln-treated cells were stained using the anti-LC3 Alexa Fluor^®^ 555 conjugated antibody. The fluorescence of LC3 in the autophagosomes was quantified using the Muse^®^ Cell Analyzer.

### 2.8. Annexin V Assay

Apoptotic cells were detected using the Muse^®^ Annexin V & Dead Cell Kit (Luminex, Austin, TX, USA). CT26 (5 × 10^3^ cells/well) and HCT116 cells (1 × 10^4^ cells/well) were spread in a 96-well culture plate and stabilized overnight. After Aln (20–80 μM) treatment for 24 h, the cells were collected (1 × 10^6^ cells/mL), and 100 μL of mixed cells was combined with Muse^TM^ Annexin V & Dead Cell Reagent. The samples were stained for at least 20 min with the light blocked. The Muse^®^ Cell Analyzer detected apoptotic cells in Aln-treated CRC cells.

### 2.9. Mitopotential Assay

The Muse^®^ Mitopotential Kit (Luminex, Austin, TX, USA) was utilized to assess Aln-treated CRC cells. CT26 and HCT116 cells (1 × 10^4^ cells/well) were spread in a 96-well culture plate and treated with Aln (20–80 μM) for 24 and 48 h, respectively. Aln-treated cells were harvested and mixed with the mitopotential working solution containing the MitoPotential Dye for at least 20 min in a 37 °C CO_2_ incubator. The samples were mixed with 7-AAD and incubated for 5 min. The Muse^®^ Cell Analyzer measured the depolarized rate of cells.

### 2.10. Western Blot

Cells (3 × 10^5^ cells/well) were spread on a 6-well culture plate and incubated overnight. Aln (20–80 μM) was used to treat cells for 24 h and 48 h, respectively. The cells and lung tissues collected from the in vivo experiments underwent lysis with PRO-PREP protein extraction solution (iNtRon Biotechnology, Seoul, Republic of Korea) to obtain the total proteins. Cell or tissue lysates were collected and vortexed every 10 min for 50 min. They were centrifuged at 13,000 rpm for 10 min to obtain the supernatant. The lysates were mixed with 4X sample buffer and subjected to boiling at 95 °C. After 5 min, the proteins underwent separation by utilizing sodium dodecyl sulfate-polyacrylamide gel electrophoresis, followed by transfer to polyvinylidene fluoride membranes. A blocking process was conducted with the membranes and EveryBlot blocking buffer (Bio-Rad Laboratories, Hercules, CA, USA) for 5 min. These membranes were incubated overnight at 4 °C with primary antibodies and washed with 0.1% tween 20 in TBS (TBST) for 45 min. The membranes were incubated with secondary antibodies for 60 min and then washed at 15 min intervals for 2 h. The detection of protein bands was facilitated using the ECL solution. The protein bands were visualized utilizing Alliance Q9 Advanced (UVITEC, Cambridge, UK).

### 2.11. Real-Time Reverse Transcription Polymerase Chain Reaction (RT-PCR)

The RNA-spin^TM^ Total RNA Extraction Kit (iNtRon Biotech, Seoul, Republic of Korea) and the iScript cDNA Synthesis Kit (Bio-Rad Laboratories, Hercules, CA, USA) were employed in compliance with the specified protocol for total RNA extraction and cDNA synthesis. CT26 and HCT116 (5 × 10^5^ cells/well) cells were spread on a 6-well culture plate and treated with Aln (20–80 μM) for 24 h. Subsequently, 350 μL of R-buffer was added to the cells for 20 min. A total of 350 μL of 70% ethanol was added to the lysate and centrifuged at 13,000 rpm for 30 s. The washing buffer was added and centrifuged at 13,000 rpm for 30 s. The final lysates were centrifuged for 2 min at 13,000 rpm to dry the column membrane. Subsequently, the elution buffer was added for 5 min and centrifuged at 13,000 rpm for 1 min. After quantifying the total RNA, the processes for priming, reverse transcription, and RT inactivation were executed at 25 °C for 5 min, 46 °C for 20 min, and 95 °C for 1 min, respectively. Real-time PCR reactions were conducted utilizing Real-Time qPCR 2X Master Mix (ElpisBiotech, Daejeon, Republic of Korea) and the StepOnePlus^TM^ Real-time PCR System (Applied Biosystems by Thermo Fisher Scientific, Waltham, MA, USA). The thermal cycling conditions were established as follows: 1 cycle for pre-incubation at 95 °C for 10 min, followed by 40 cycles for amplification at 95 °C for 15 s and 60 °C for 1 min. Melting curve analysis was carried out during the last phase by increasing the temperature from 60 °C for 1 min to 95 °C for 15 s. The following sequences of the primers were used for the mouse genes: lc3b 5′-CGTCCTGGACAAGACCAAGT-3′ (forward) and 5′-ATTGCTGTCCCGAATGTCTC-3′ (reverse); p62 5′-TGTGGAACATGGAGGGAAGAG-3′ (forward) and 5′-TGTGCCTGTGCTGGAACTTTC-3′ (reverse); β-actin 5′-GACAGATGCAGAAGGAGATTACT-3′ (forward) and 5′-TGATCCACATCTGCTGGAAGGT-3′ (reverse). The following sequences of the primers were used for the human genes: LC3B 5′-GAGAAGCAGCTTCCTGTTCTGG-3′ (forward) and 5′-GTGTCCGTTCACCAACAGGAAG-3′ (reverse); p62 5′-GCACCCCAATGTGATCTGC-3′ (forward) and 5′-CGCTACACAAGTCGTAGTCTGG-3′ (reverse); GAPDH 5′-CATGAGAAGTATGACAACAGCCT-3′ (forward) and 5′-AGTCCTTCCACGATACCAAAGT-3′ (reverse).

### 2.12. Lung Metastatic Mouse Model

For the experimental mouse model of lung metastasis, CT26 cells (2 × 10^5^ cells/100 μL) were injected intravenously through the tail vein. Aln (3.125, 6.25, and 12.5 mg/kg) was administered orally once daily for 2 weeks. Moreover, 5-FU (10 mg/kg) was inserted intraperitoneally in the positive control group once every 3 days for 2 weeks. After completion of the administration period, the mice were euthanized, and serum samples were obtained for serological analysis. To count the number of nodules, lung tissue was stained and fixed with Bouin’s solution. Additionally, lung tissues were excised, washed with PBS, and kept at −80 °C for protein analysis. All data were double-blind tested.

### 2.13. Statistical Analysis

The data were expressed as the mean ± standard deviation (S.D.), and statistical evaluations were performed using the Student’s *t*-test and one-way ANOVA to discern group variations. All statistical analyses were executed utilizing SPSS Statistics version 26 (IBM Corporation, Armonk, NY, USA). Values with * *p* < 0.05, ** *p* < 0.01, and *** *p* < 0.001 compared to the control group indicated a statistically significant difference.

## 3. Results

### 3.1. The Effect of Aln on the Cell Viability of CRC Cells

**Figure 1:**
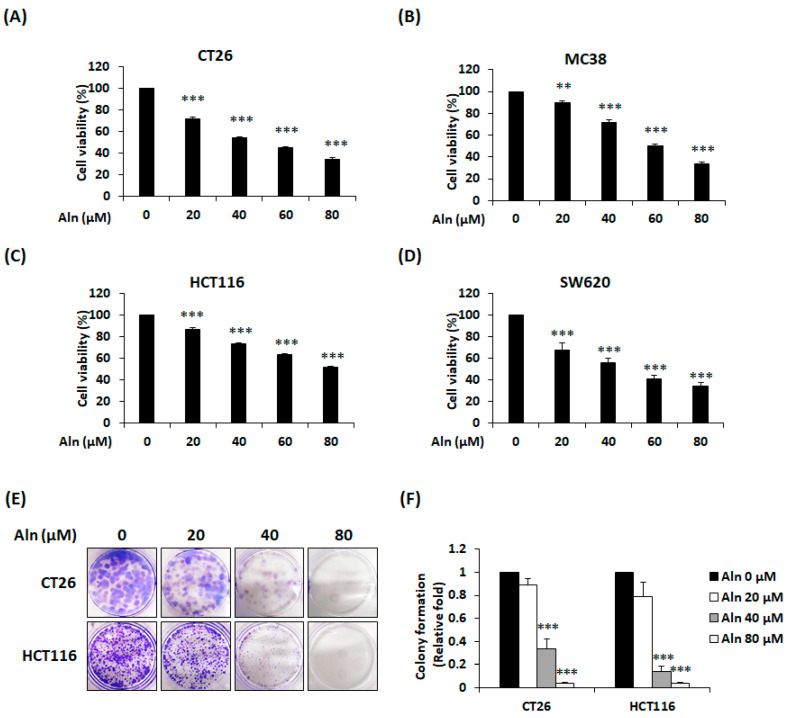
Aln inhibits cell viability and colony forming ability of CRC cells. Cell viability of Aln-treated CT26 (**A**), MC38 (**B**), HCT116 (**C**), and SW620 (**D**) cells. Cell viability was assessed using CCK solution. (**E**) Colonies of CT26 and HCT116 cells 7 days after Aln treatment. (**F**) Relative colony formation levels. All values are mean ± S.D. from three independent experiments. ** *p* < 0.01 and *** *p* < 0.001.

To confirm the cytotoxic activity of Aln on CRC cell lines, cell viability was investigated using CT26, MC38, HCT116, and SW620 cells. Aln (20–80 μM) treatment for 24 h diminished the viability of four CRC cell lines in proportion to the concentration (Figure 1A–D). In particular, Aln markedly reduced cell viability by 34.81% ± 1.10% in CT26 cells, 33.45% ± 1.98% in MC38 cells, 51.91% ± 1.88% in HCT116 cells, and 34.25% ± 2.94% in SW620 cells at the highest concentration of 80 μM. The IC50 values were 54.31 ± 0.80 μM for CT26 cells, 62.06 ± 1.65 μM for MC38 cells, 85.99 ± 1.03 μM for HCT116 cells, and 52.26 ± 11.73 μM for SW620 cells. Next, a colony formation assay was carried out to discover whether Aln can inhibit colony formation and proliferation of CRC cells. As presented in Figure 1E,F, Aln treatment for 7 days markedly suppressed the colony growth ability of both types of CRC cells.

### 3.2. Effect of Aln on Cell Cycle Arrest of CRC Cells

**Figure 2:**
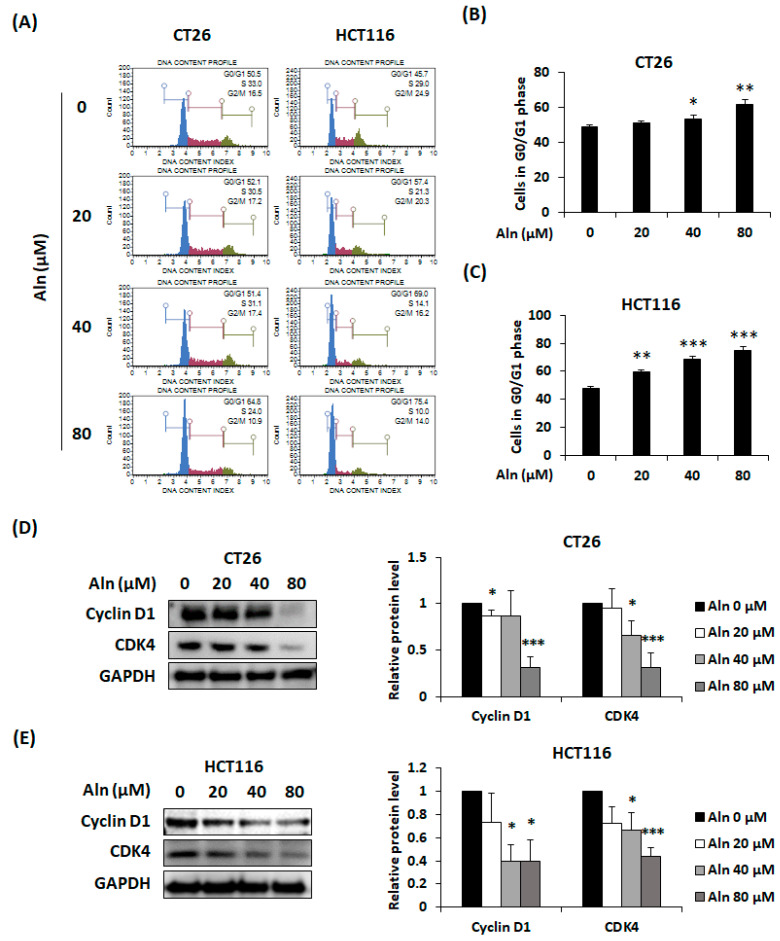
Aln induces G0/G1 phase arrest through reducing cyclin D1-CDK4 expression in CRC cells. (**A**) Cell cycle analysis of Aln-treated CT26 and HCT116 cells for 24 h. (**B**,**C**) Percentage of cells in G0/G1 phase in CT26 (**B**) and HCT116 (**C**) cells. (**D**,**E**) Cyclin D1-CDK4 protein expression in CT26 (**D**) and HCT116 (**E**) cells with Aln treatment for 24 h and 48 h, respectively. Protein density was determined by using Image J program. All values are mean ± S.D. from three independent experiments. * *p* < 0.05, ** *p* < 0.01, and *** *p* < 0.001.

Cell cycle phase detection was first analyzed to clarify the cause of the reduction in CRC cell viability due to Aln treatment. CT26 and HCT116 cells were treated with Aln (20–80 μM) for 24 h and analyzed by flow cytometry. Aln caused G0/G1 phase arrest in both types of CRC cells in a concentration-dependent manner (Figure 2A). At the highest concentration of 80 μM, Aln increased the percentage of cells belonging to G0/G1 from 49.07% ± 1.24% to 61.6% ± 2.8% in CT26 cells and from 47.67% ± 1.70% to 74.9% ± 2.88% in HCT116 cells (Figure 2B,C). Aln treatment suppressed the cyclin D1 and CDK4 protein expressions in both types of CRC cells (Figure 2D,E). Moreover, cyclin B1 and CDK1 expression also decreased due to Aln treatment in CT26 and HCT116 cells (Figure S1). Therefore, Aln led to G0/G1 phase arrest through inhibiting cyclin–CDK complex protein expression.

### 3.3. Effect of Aln on Autophagy of CRC Cells

**Figure 3:**
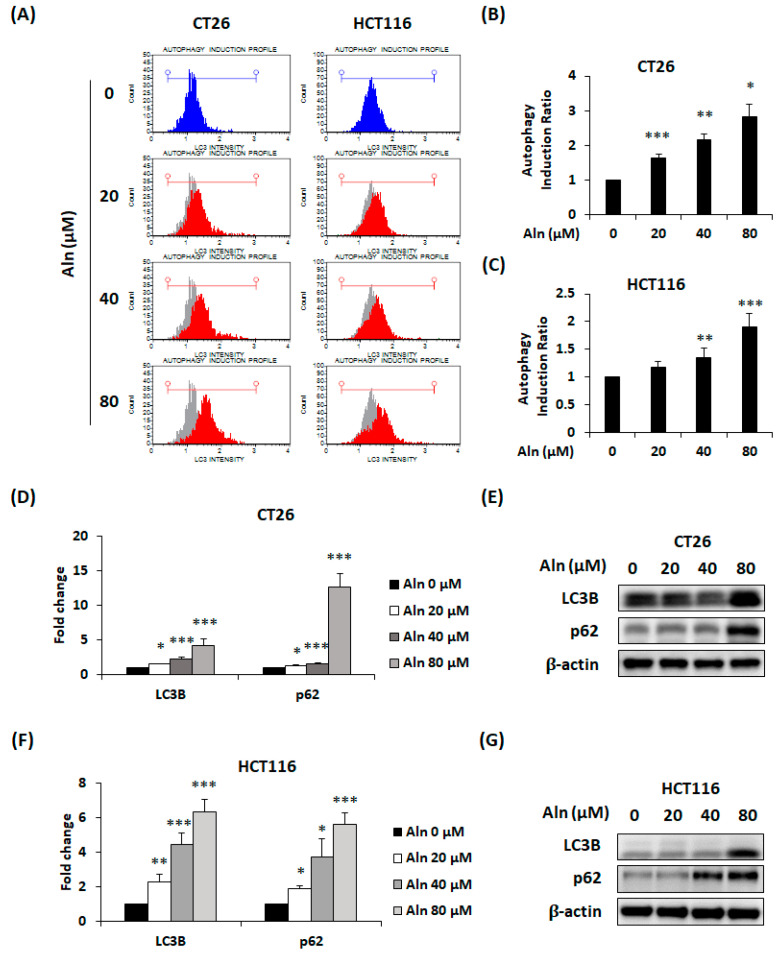
Aln increases autophagy by upregulating LC3B and p62 expression in CRC cells. (**A**) Detection of LC3 in Aln-treated CRC cells was analyzed by flow cytometry. The blue graph serves as a baseline for autophagy measurements, while the red graph represents the autophagy of cells following Aln treatment. (**B**,**C**) Autophagy induction ratio of Aln-treated CT26 (**B**) and HCT116 (**C**) cells. (**D**,**F**) mRNA expression of LC3B and p62 in CT26 (**D**) and HCT116 (**F**) cells after Aln treatment for 24 h was quantified by real-time RT-PCR. (**E**,**G**) Autophagy-related protein expression of LC3B and p62 in CT26 (**E**) and HCT116 (**G**) cells after Aln treatment for 24 and 48 h, respectively. Proteins were assayed by using Western blot. All values are mean ± S.D. from three independent experiments. * *p* < 0.05, ** *p* < 0.01, and *** *p* < 0.001.

To identify other causes of inhibition of CRC cell proliferation by Aln, an additional experiment was conducted to decide whether autophagy was induced in Aln-treated CT26 and HCT116 cells. Both types of CRC cells treated with Aln were analyzed by LC3-II detection. As a result, it was observed that Aln treatment induces autophagy of both types of CRC cells depending on the concentration (Figure 3A). The autophagy induction ratio was increased by approximately 2.8 and 2 times in CT26 and HCT116 cells, respectively, by the highest concentration of Aln (Figure 3B,C). In addition, mRNA expression of autophagy-associated factors LC3B and p62 was upregulated by Aln treatment for 24 h in CRC cells (Figure 3D,F). Additionally, LC3B and p62 protein expression levels were elevated by Aln treatment in both types of CRC cells (Figure 3E,G). Therefore, Aln can induce autophagy by augmenting LC3B and p62 mRNA and protein expression.

### 3.4. Effect of Aln on Apoptosis of CRC Cells

**Figure 4:**
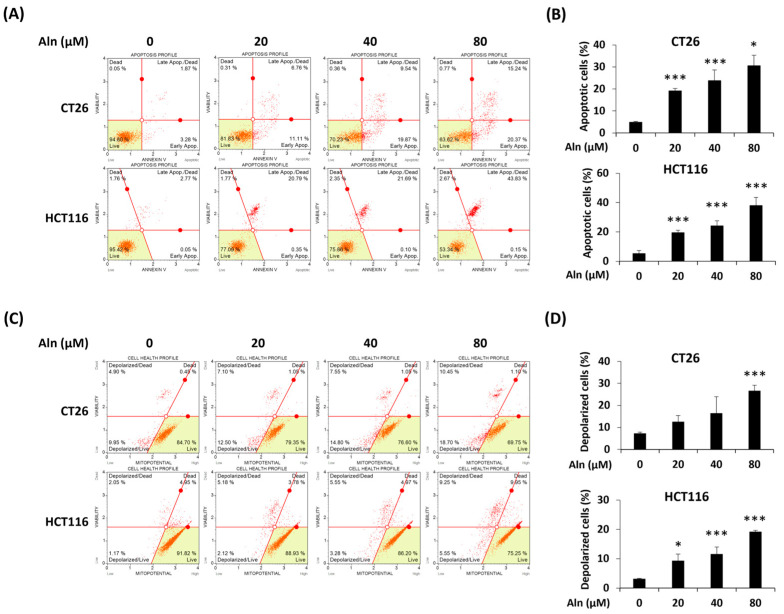
Aln promotes apoptosis through the depolarization of mitochondria in CRC cells. (**A**) Annexin V staining of CT26 and HCT116 cells treated with Aln for 24 h. The cells were analyzed by flow cytometry. Dot blot in the green area means live cells. The red solid line distinguishes the area of live cells, early apoptosis cells, late apoptosis cells, and dead cells. (**B**) The percentages of apoptotic cells in Aln-treated CT26 and HCT116 cells. (**C**) Mitochondrial depolarization of CRC cells after Aln treatment for 24 and 48 h, respectively. Flow cytometry was used to analyze the cells. The red solid line distinguishes the area of live cells, depolarized/live cells, depolarized/dead cells, and dead cells. (**D**) The percentages of depolarized cells in Aln-treated CRC cells. All values are the mean ± S.D. from three independent experiments. * *p* < 0.05 and *** *p* < 0.001.

Annexin V staining was conducted to determine whether Aln can promote apoptosis of CRC cells. After Aln treatment, apoptotic cells increased in a concentration-related manner (Figure 4A). In particular, 80 μM of Aln significantly augmented the apoptosis rates of CT26 and HCT116 cells by 25.62% ± 4.76% and 32.73% ± 5.50%, respectively (Figure 4B). To certify that Aln can promote apoptosis of CRC cells through changing the mitochondrial transmembrane potential, the mitochondrial membrane potential was analyzed in CT26 and HCT116 cells. Mitochondrial depolarization of CRC cells was increased by Aln treatment (Figure 4C). At the highest concentration of Aln, the proportion of depolarized cells increased by about 19.38% ± 2.52% in CT26 cells and 16.04% ± 0.44% in HCT116 cells compared to the control group (Figure 4D). Therefore, Aln can induce mitochondrial-mediated apoptosis of CRC cells.

### 3.5. Effect of Aln on Apoptosis-Related Factors’ Expression in CRC Cells

**Figure 5:**
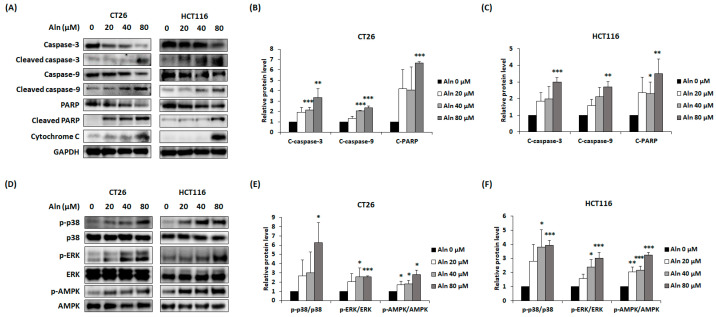
Aln regulates apoptosis-related proteins by phosphorylating p38, ERK, and AMPK in CRC cells. (**A**–**C**) Apoptotic-associated proteins in CT26 and HCT116 cells after Aln treatment for 24 and 48 h, respectively. (**D**–**F**) Phosphorylation of p38, ERK, and AMPK in CRC cells after Aln treatment for 30 min following 4 h of starvation. Protein density was measured using Image J program. All values are mean ± S.D. from three independent experiments. * *p* < 0.05, ** *p* < 0.01, and *** *p* < 0.001.

To further understand whether Aln can change the expression of apoptosis-associated proteins, Western blotting was performed using Aln-treated CT26 and HCT116 cells. Aln promoted caspase-3, caspase-9, and PARP cleavage, whereas cytochrome C expression was increased by Aln treatment in both types of CRC cells (Figure 5A–C). To explore the signaling pathways related to Aln-mediated suppression of cell proliferation, Western blotting was performed on CRC cells exposed to Aln for a short time. Aln increased phosphorylation of AMPK, p38, and ERK, whereas phosphorylation of JNK was not changed in either type of CRC cells (Figure 5D). At 80 μM of Aln, the phosphorylated expression of AMPK increased by about 2.8 times in CT26 cells and 3.2 times in HCT116 cells. Additionally, Aln increased the phosphorylation of p38 by about 6 times in CT26 cells and 3.9 times in HCT116 cells at a concentration of 80 μM. Furthermore, Aln (80 μM) treatment increased the phosphorylation of ERK by about 2.5 times in CT26 cells and 3 times in HCT116 cells (Figure 5D,F).

### 3.6. Effect of Aln on Lung Metastasis of CT26 Cells in Mouse Model

**Figure 6:**
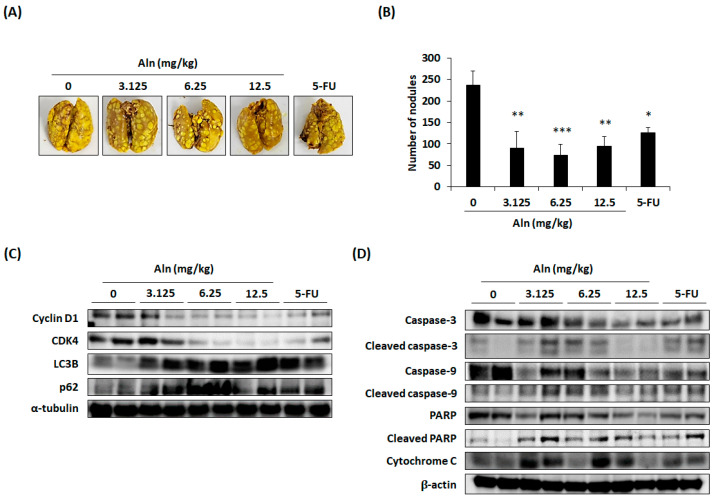
Aln attenuates pulmonary metastasis of CT26 cells by promoting cell cycle arrest, autophagy, and apoptosis in mouse models. (**A**) Representative images of lung tissues 14 days after the in vivo experiment. The lung tissue was fixed and dyed with Bouin’s solution. (**B**) The number of tumor nodules. (**C**) Cell cycle proteins and autophagy-associated proteins in lung tissues. (**D**) Apoptotic-associated protein expression in lung tissues after oral administration of Aln for 14 days. The proteins were identified by Western blot analysis. All values are the mean ± S.D. from three independent experiments. * *p* < 0.05, ** *p* < 0.01, and *** *p* < 0.001.

To determine whether Aln can restrict the ability of CRC cells to metastasize to the lungs, an animal experiment was carried out. The number of lung nodules was significantly diminished by the oral administration of Aln (3.125, 6.25, and 12.5 mg/kg) for 2 weeks (Figure 6A). Lung metastasis was reduced by about 46.40% with the intraperitoneal injection of 5-FU (10 mg/kg), used as a positive control (Figure 6B). The liver function parameters (AST and ALT) and kidney function parameters (creatinine and BUN) in both the Aln-treated groups and the 5-FU group exhibited no significant deviations compared to those of the control group, as detailed in Table 1.

To further explore whether Aln can engender cell cycle arrest, autophagy, and apoptosis in mouse models, changes in the expression levels of relevant proteins in lung tissues were analyzed. Cyclin D1-CDK4 expression diminished in all Aln-administered groups and in the 5-FU group. Also, Aln treatment elevated the autophagy-related proteins p62 and LC3B (Figure 6C). Both Aln and 5-FU groups were found to stimulate caspase-3, caspase-9, and PARP cleavage, with an elevation in cytochrome C protein expression (Figure 6D). This result substantiates the observed effect in the outcomes of the in vitro experiment. Consequently, Aln prevented pulmonary metastasis of CRC cells by causing cell cycle arrest, autophagy, and apoptosis in a mouse model.

## 4. Discussion

Aln is a nonphenolic diarylheptanoid isolated from *A. katsumadae* seeds, *Alnus pendula*, and *Curcuma xanthorrhiza* [18,19,23]. *A. katsumadae* is a medicinal plant that has been utilized for a long time in East Asia. In particular, mature seeds of *A. katsumadae* are used medicinally after pericarpium removal [24]. It has been applied for vomiting, retching, abdominal pain, diarrhea, belching, and anorexia in oriental medicine [17]. Moreover, prior studies have reported its pharmacological properties such as anti-inflammatory, anti-asthmatic, antioxidant, antimicrobial, and anti-emetic effects [24,25,26,27,28]. In addition, *A. katsumadae* ethanol extract which contains Aln can suppress the viability of Panc-28 pancreatic cancer cells and the tumor growth of A549 lung cancer cells in xenograft mouse models [29].

Regarding the anti-cancer effect of Aln, recent investigations have unveiled its effect on suppressing liver cancer cell line proliferation by modulating the PI3K/Akt/mTOR signaling pathway [22]. Aln exhibited apoptosis-mediated cellular death of human hepatocellular carcinoma cells HepG2 and BEL-7402 at doses of 50 μM and 70 μM, respectively. In addition, Aln did not induce cytotoxicity on human normal cells such as HUVEC and HaCaT after treatment at the maximum concentration of 100 μM for 48 h [22]. In this study, 20–80 μM of Aln leads to a significant reduction in the viability of four highly metastatic CRC cell lines within 24 h. As a result of exploring the cause of Aln-induced cell death, Aln promoted arrest in the G0/G1 phase through inhibiting cyclin–CDK complex protein expression. Moreover, autophagy occurred by upregulating LC3B and p62 mRNA and protein expression in CRC cells after Aln treatment. In the intact autophagy process, an autophagic substrate p62 undergoes degradation by lysosomal proteases in autolysosomes [30]. However, in the event of an impaired autophagy process, wherein autophagosomes are not degraded, p62 may accumulate, potentially leading to cell death [31,32]. Nevertheless, it can be concluded that the crucial cause of CRC cell death was apoptosis accompanied by mitochondrial depolarization. Aln increased the apoptosis rates of CT26 and HCT116 cells by up to 25.62% and 32.73%, respectively, and induced the activation of apoptosis-involved proteins in proportion to the concentration.

AMPK is activated in various metabolic stress situations and helps to preserve cellular energy balance and stability [33]. AMPK activation can trigger cell cycle arrest, autophagy, and apoptosis, functioning as a tumor inhibitor in cancer cells [34]. The MAPK signaling pathway consists of p38, ERK, and JNK. It is engaged in diverse cellular processes such as proliferation, differentiation, apoptotic events, autophagy, survival, death, and inflammation [35]. In cancer, alterations in MAPK signaling cascades are found in response to genetic and epigenetic changes [36]. The p38 and ERK pathways have been largely recognized as survival signaling cascades and play roles in several steps of tumor development. Numerous investigations have demonstrated that cancer cell death can be targeted in a way that depends on MAPK pathway activation [37,38]. A study reported that quercetin promoted apoptosis by increasing mitochondrial depolarization and intracellular ROS generation by activating the AMPK/p38 pathway in HCT116 cells [39]. Similarly, oleanolic acid induced the phosphorylation of p38, ERK, and JNK, resulting in caspases and PARP cleavage, as well as the release of cytochrome C. Furthermore, this study confirmed that the apoptosis induced by oleanolic acid is due to the activation of p38 signaling pathways in several types of tumor cells [40]. Moreover, the MAPKs (p38, ERK, and JNK) are constitutively phosphorylated in physalin B-treated HCT116 cells and partially mediate the autophagic response and apoptosis [41]. In this study, we proved that Aln upregulated the phosphorylation of AMPK, p38, and ERK in CRC cells.

To conduct an in vivo experiment, a scientific literature search was needed to establish the appropriate concentrations. Intraperitoneal injection of Aln (25–100 mg/kg) in BALB/c nude mice one time a day for 14 days resulted in insignificant changes in body weight and lower toxicity compared to the 5-FU group [22]. A total of 10 mg/kg of Aln was administered intraperitoneally to diet-induced obese C57BL/6J mice once daily for 7 days [42]. In addition, subcutaneous injection of Aln (25 mg/kg) showed a protective effect on pneumonia caused by *Streptococcus pneumoniae* in BALB/c mice [21]. Based on these studies, the maximum concentration of Aln 12.5 mg/kg for oral administration was used on BALB/c mice for 14 days in this experiment. Oral administration of Aln for 2 weeks showed a suppressive effect on lung metastasis of CRC cells compared to the control. Aln at 3.125 and 6.25 mg/kg showed a dose-dependent inhibitory effect, but the highest concentration of 12.5 mg/kg showed a lower inhibitory effect than other low-concentration groups. Hence, it is crucial to identify the optimal dosage for utilizing Aln in clinical therapeutic applications. p62 and LC3B expression levels were elevated, while the expression of cyclin D1-CDK4 was diminished. Additionally, the activation of PARP and caspase-3 was promoted in the Aln-administered groups in comparison with the control group. Furthermore, cleavage of caspase-9 and cytochrome C expression was increased, which indicates mitochondria-mediated apoptosis. Based on these results, Aln suppressed the development of pulmonary metastasis of CT26 cells and the observed effect can be attributed to the promotion of cell cycle arrest, autophagy, and apoptosis, consistent with in vitro experiments.

## 5. Conclusions

This study provides compelling evidence that Aln can significantly restrict the proliferative activity and metastatic ability of CRC cells. These effects are achieved utilizing cell cycle arrest, autophagy induction, and mitochondria-mediated apoptosis, facilitated by the activation of the AMPK and p38/ERK signaling pathways. Consequently, this study implies that Aln could hold potential as a new therapeutic option for CRC.

## Figures and Tables

**Table 1:** Serum analysis results of AST, ALT, creatinine, and BUN in lung metastasis mouse model.

	Aln (mg/kg)				5-FU(10 mg/kg)
	0	3.125	6.25	12.5	
AST (IU/L)	199.75 ± 117.25	106.17 ± 49.95	143.14 ± 62.82	182.0 ± 38.24	143.5 ± 45.93
ALT (IU/L)	30.2 ± 13.39	19.33 ± 3.98	24.33 ± 5.79	22.8 ± 3.35	22.0 ± 5.70
Creatinine (mg/dL)	0.13 ± 0.03	0.12 ± 0.01	0.09 ± 0.03	0.13 ± 0.05	0.08 ± 0.01
BUN (mg/dL)	18.83 ± 1.83	17.29 ± 3.45	16.29 ± 1.80	17.86 ± 2.48	19.33 ± 2.80

## Data Availability

The original contributions presented in this study are included in the article; further inquiries can be directed to the corresponding authors.

## Supplementary Materials

The following supporting information can be downloaded at: https://www.mdpi.com/article/10.3390/nu16213737/s1.
