# Supplementary material for: Inhibitory Effect of Alnustone on Survival and Lung Metastasis of Colorectal Cancer Cells
Source: Nutrients. 2024 Oct 31;16(21):3737. doi: 10.3390/nu16213737 (PMC11547205; doi:10.3390/nu16213737)
Supplement: Supplementary file 1 [file nutrients-16-03737-s001.zip › nutrients-3208771-supplementary.pdf]

Supplementary figure 1.

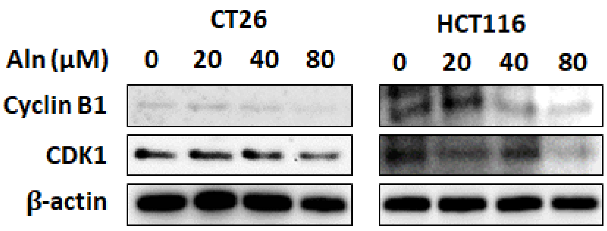

Supplementary figure 1. Aln induces cell cycle arrest by decreasing the expression of cyclin B1 and CDK1 in CRC cells. Cyclin B1 and CDK1 protein expression in CT26 and HCT116 cells wit h Aln treatment for 24 h and 48 h, respectively. β-actin was used as a loading control.
